# Design of Topical Ocular Ciprofloxacin Nanoemulsion for the Management of Bacterial Keratitis

**DOI:** 10.3390/ph14030210

**Published:** 2021-03-03

**Authors:** Ahmed Adel Ali Youssef, Chuntian Cai, Narendar Dudhipala, Soumyajit Majumdar

**Affiliations:** 1 Department of Pharmaceutical Technology, Faculty of Pharmacy, Kafrelsheikh University, Kafrelsheikh 33516, Egypt; aayousse@go.olemiss.edu; 2 Department of Pharmaceutics and Drug Delivery, School of Pharmacy, University of Mississippi, Oxford, MS 38677, USA; ccai@go.olemiss.edu (C.C.); ndudhipa@olemiss.edu (N.D.); 3 Research Institute of Pharmaceutical Sciences, University of Mississippi, Oxford, MS 38677, USA

**Keywords:** bacterial keratitis, ciprofloxacin, nanoemulsion, stability, transcorneal permeability, surfactant, oleic acid, Labrafac^®^ Lipophile WL 1349

## Abstract

Bacterial keratitis (BK) is a critical ocular infection that can lead to serious visual disability. Ciprofloxacin (CIP), moxifloxacin (MOX), and levofloxacin (LFX) have been accepted as monotherapies by the US Food and Drug Administration for BK treatment. CIP is available commercially at 0.3% *w*/*v* concentration as an ophthalmic solution and as an ointment for ocular delivery. Because of solubility issues at physiological pH, CIP precipitation can occur at the corneal surface post instillation of the solution dosage form. Consequently, the ocular bioavailability of CIP is reduced. The ointment dosage form is associated with side effects such as blurred vision, itching, redness, eye discomfort, and eye dryness. This study aimed to design a CIP loaded nanoemulsion (NE; CIP-NE) to facilitate drug penetration into the corneal layers for improved therapeutic outcomes as well as to overcome the drawbacks of the current commercial ophthalmic formulations. CIP-NE formulations were prepared by hot homogenization and ultrasonication, using oleic acid (CIP-O-NE) and Labrafac^®^ Lipophile WL 1349 (CIP-L-NE) as the oily phase, and Tween^®^ 80 and Poloxamer 188 as surfactants. Optimized CIP-NE was further evaluated with respect to in vitro release, ex vivo transcorneal permeation, and moist heat sterilization process, using commercial CIP ophthalmic solution as a control. Optimized CIP-O-NE formulation showed a globule size, polydispersity index, and zeta potential of 121.6 ± 1.5 nm, 0.13 ± 0.01, and −35.1 ± 2.1 mV, respectively, with 100.1 ± 2.0% drug content and was spherical in shape. In vitro release and ex vivo transcorneal permeation studies exhibited sustained release and a 2.1-fold permeation enhancement, respectively, compared with commercial CIP ophthalmic solution. Autoclaved CIP-O-NE formulation was found to be stable for one month (last time-point tested) at refrigerated and room temperature. Therefore, CIP-NE formulation could serve as an effective delivery system for CIP and could improve treatment outcomes in BK.

## 1. Introduction

Out of the estimated 30,000 cases of microbial (parasitic, bacterial, and fungal) keratitis that occurs annually in the USA, the more common pathogens are bacterial in nature [1,2]. Bacterial infections constitute a major disease class for the eye and include conjunctivitis, endophthalmitis, keratitis, blepharitis, dacryocystitis, and orbital cellulitis [3]. Ophthalmic infections can lead to damages that could lead to vision loss if left uncontrolled without proper treatment [4]. Bacterial keratitis (BK) is a destructive ocular infection that occurs when the corneal epithelium is compromised due to injury, leading to ulceration and inflammation [4]. Gram-positive *Staphylococcus aureus*, *Staphylococcus epidermidis,* and numerous *Streptococcus* and *Bacillus* species, in addition to Gram-negative bacteria such as *Moraxella lacunata*, *Pseudomonas aeruginosa*, *Serratia marcescens*, *Haemophilus influenzae* and *Microbacterium liquefaciens* are the leading pathogens isolated in BK [3]. Monotherapy with fluoroquinolones, such as ciprofloxacin (CIP), moxifloxacin (MOX), levofloxacin (LFX) and ofloxacin (OFX), is the first choice for the treatment of BK in the United States [4]. Effective early treatment of BK can avoid progression to corneal perforation, endophthalmitis, or blindness [4,5].

CIP is a second-generation broad-spectrum fluoroquinolone bactericidal antibiotic. It targets two important bacterial topoisomerase enzymes involved in DNA synthesis, DNA gyrase and DNA topoisomerase IV. CIP is active against many aerobic Gram-positive and Gram-negative bacteria, so it has a good therapeutic activity in the treatment of a wide variety of bacterial infections including ocular bacterial infections [6]. Thus, CIP is one of the first choices in the treatment of BK [7], endophthalmitis [8], bacterial and allergic conjunctivitis [9], and for many other bacterial infections of the eye [10]. The commercially available CIP ophthalmic solution needs frequent administration due to CIP’s poor ocular bioavailability. The solubility of CIP being low at tear fluid pH, precipitation takes place on administration of the commercial ophthalmic solutions, leading to low ocular bioavailability as well as ocular irritation [11].

Kowalski et al. recorded the MIC90s for CIP, OFX, LFX, gatifloxacin (GFX), and MOX against 177 bacterial keratitis isolates [12]. They reported that, especially for fluoroquinolone-resistant *Staphylococcus aureus*, the MIC90s for Gram-positive bacteria were significantly lower for fluoroquinolones of the fourth generation than second generation—3 µg/mL for MOX and GFX versus 64 µg/mL for LFX, CIP, and OFX. However, CIP was still superior against Gram-negative bacteria, especially *Pseudomonas aeruginosa,* compared to other fluoroquinolones (0.125 µg/mL for CIP, 0.5 µg/mL for LFX, 0.38 µg/mL for GFX, and 0.75 µg/mL for MOX). Similar outcomes were also reported by Oliveira et al. [13]. Based on these earlier studies, and the fact that *Pseudomonas aeruginosa* is known to cause severe cases of BK and corresponding corneal blindness, CIP was selected for this study [14].

Topical ocular delivery is the most popular non-invasive route for the treatment of ocular diseases because of ease of application, and patient compliance [15]. However, precorneal elimination due to nasolacrimal drainage and high tear fluid turnover continues to be the major drawback for topical application [16]. Only 1–5% of the active moiety applied to the ocular surface reaches the deeper intraocular tissues while the remaining amount overflows from the conjunctival sac and/or is lost through the conjunctiva and nasolacrimal drainage and could produce systemic side effects [15,16]. Various techniques to improve ocular bioavailability of drugs such as the addition of mucoadhesive agents [17] and viscosity enhancers in liquid dosage forms, prodrugs [18,19], ion-exchange resin-based formulations [20], films [21], emulsions [22], implants [23], in-situ gelling systems [24,25], and controlled systems, such as ocular inserts [26], have been explored in an effort to overcome the many drawbacks associated with conventional ocular drug delivery systems. These strategies aim to improve retention at the ocular surface or increase transcorneal penetration, or both.

Nano-sized carriers are efficient drug delivery systems for lipophilic drugs, which comprise about 40% of newly discovered active pharmaceutical ingredients [27]. Nanoemulsion (NEs) are submicron-sized thermodynamically stable isotropic systems with a globule size that typically falls between 20–200 nm [28]. NE formulations have been commonly used in the development of pharmaceutical platforms for topical, intravenous, ocular, and other routes of drug delivery [29]. Moreover, NE formulations have been tested for increasing ocular bioavailability [30], enhancing drug stability [31], and sustaining the release of ophthalmic drugs [32]. NE formulations are easy to prepare, scale-up, and many excipients have been reported and recognized to be safe for their preparation [33].

Although the combination of CIP eye drops by day and ointment application at night was established as a strategy for prolonging the antibacterial action, the ointment is difficult to apply, and is known to cause ocular adverse effects such as irritation, interference with vision, and redness of the eye [15]. Additionally, increased frequency of administration increases systemic drug absorption which may lead to systemic complications [15].

Sai et al. have reported that transcorneal flux (~2.9-fold) and ocular distribution of CIP were enhanced from melt-cast polymeric inserts when compared to the control formulation [34]. Mostafa et al. also reported in earlier studies that CIP-loaded liposomal formulations achieved almost 4-folds enhancement in ocular bioavailability compared to commercial CIP eye drops [35]. However, liposomes suffer from physicochemical stability issues due to aggregation, fusion, degradation, hydrolysis, and phospholipid oxidation [36,37]. In another study, Murthy et al. succeeded in delivering CIP hydrochloride to the anterior chamber of the eye by applying transcorneal iontophoresis; however, many histopathological changes have been reported after transcorneal iontophoresis such as hemorrhagic necrosis, conjunctival edema, and infiltration of polymorphonuclear cells [38,39].

In the current research project, in search for alternative strategies for CIP delivery, NEs were evaluated because they are known to improve ocular drug bioavailability, are retained on the ocular surface, and facilitates the distribution of drugs to the deeper ocular tissues [40]. A review of the literature did not yield any reports on CIP-NE formulations for ocular delivery. Hence, an attempt was made to develop an alternative delivery system, which would help CIP remain in the solubilized state at physiological pH and increase drug permeation into the corneal layers for improved therapeutic outcomes in BK treatment. The objective of the current research was to overcome the challenges encountered with topical ocular administration of CIP by developing CIP-NE formulations. The optimized CIP-NE formulation was further evaluated for in vitro release and ex vivo corneal permeation in comparison with commercial eye drops.

## 2. Results and Discussion

### 2.1. Screening of Oils

Oil-in-water (*o*/*w*) type NE formulations are the best choice for lipophilic drugs. NEs have been widely investigated for their use in ocular drug delivery. Moreover, there are a number of NE dosage forms available either in the market, such as Restasis^®^ and Cyclokat^®^ that are used for dry eye disease, or within clinical trials such as Brimonidine Tartrate eye drops for dry eye disease under phase ΙΙΙ [41,42]. Drug loading is a vital parameter in the development of NE formulations for hydrophobic drugs and is highly dependent on the drug’s solubility in the formulation components such as oils and surfactants. However, the solubility of the drug in the oil phase, which constitutes the dispersed phase in the *o*/*w* emulsion, is most important. If the surfactant or cosurfactant, used to stabilize the emulsion, helps in drug solubilization through micelle formation, there could be a risk of drug precipitation as the dilution of the NE formulation would lower the solubilizing capacity of the surfactant or cosurfactant [43,44]. Therefore, oil is considered the crucial excipient in the preparation of NE formulations. Oil screening results are represented in Table 1. Based on the observations, Labrafac^®^ Lipophile WL 1349 (LWL) and oleic acid (OA) were selected for the preparation of CIP-L-NE and CIP-O-NE, respectively.

### 2.2. Preparation and Physical Characterization of CIP-NE Formulations

The composition of different CIP-NE formulations is presented in Table 2. CIP-NE formulations were prepared using OA and LWL as the oily phase, selected based on the solubility of CIP in different oils: surfactants (Poloxamer 188 and Tween^®^ 80) and glycerin represented the aqueous phase. The NE formulations were optimized based on two different oils (OA and LWL), surfactant concentration (Tween^®^ 80) for each oil, drug loading (0.1–0.3% *w*/*v*) within each oil, and variation in homogenization speed with and without sonication for OA and LWL-containing placebo NE formulations with 2% *w*/*v* Tween^®^ 80.

**Figure 1:**
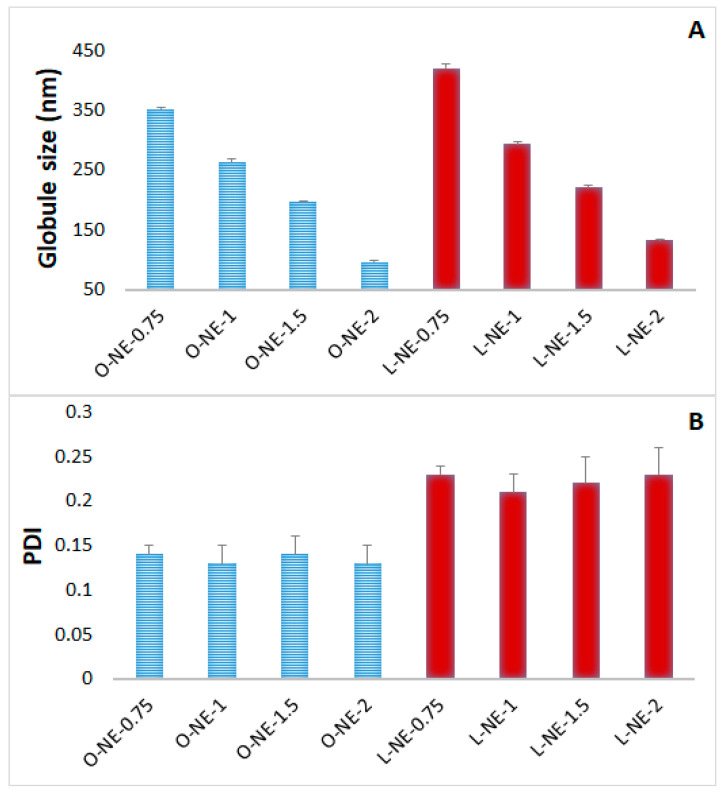
Effect of Tween^®^ 80 concentrations on (**A**) globule size, and (**B**) polydispersity index of placebo nanoemulsion formulations at homogenization speed of 11,000 rpm for 5 min and probe sonication for 10 min (mean ± SD, *n* = 3).

Effect of Tween^®^ 80 concentration: Tween^®^ 80 is the primary surfactant in the development of CIP-NE formulations. As per FDA inactive ingredient database, Tween^®^ 80 up to 4% *w*/*v* is approved for ophthalmic NE formulations. Therefore, the effect of Tween^®^ 80 concentration on NE formulation was studied. Results of varying concentrations of Tween^®^ 80 in NE placebo formulations are depicted in Figure 1. Increasing Tween^®^ 80 concentrations from 0.75% to 2% *w*/*v* decreased GS significantly (*p* < 0.05) from 351.8 to 96.3 nm and from 420.6 to 132.2 nm for OA and LWL based NE placebo formulations, respectively. However, there was no significant change in the PDI. Similar outcomes have been reported in earlier studies with NEs [45].

Effect of process parameters: The effect of process parameters on NE placebo formulations is shown in Table 3. Increasing homogenization speed (9000 to 11,000 rpm) for 5 min decreased GS from 741.3 to 395.2 nm and from 1098.6 to 488.2 nm, and it also caused a significant decrease in PDI from 0.61 to 0.43 and from 0.92 to 0.59 for OA and LWL based NE placebo formulations, respectively. Ten minutes sonication with a 10 s pulse ON and 10 s pulse OFF after homogenization at 11,000 rpm for 5 min decreased GS significantly from 395.2 ± 32.7 to 96.3 ± 2.5 nm and from 488.2 ± 70.4 to 132.2 ± 1.9 nm, there was also a significant change in PDI from 0.43 ± 0.05 to 0.13 ± 0.02 and from 0.59 ± 0.02 to 0.23 ± 0.03 for OA and LWL based NE placebo formulations, respectively. Based on these observations, a processing condition of 11,000 rpm for 5 min coupled with sonication at 40% amplitude for 10 min with a 10 s pulse ON and 10 s pulse OFF was chosen to prepare the optimized formulation.

Increasing surfactant concentration had a significant effect on GS which may be attributed to a significant decrease in surface tension and surface free energy that was produced due to high shearing conditions during homogenization [11]. The increase in homogenization speed from 9000 to 11,000 rpm provided a simultaneous increase in the breaking energy, resulting in smaller emulsion globules and thus a narrow particle size distribution [11]. A decrease in globule size by ultrasonication may be due to high energy waves that generate turbulence (due to cavitation) which breaks macroemulsion drops into smaller droplets, and it also has been reported to result in narrow size distribution [45]. The size of the nanoparticles (NPs) is important for the adhesion and interaction with the biological cell. Smaller GS (100–200 nm) can be transported by receptor mediated endocytosis uptake [10].

*Effect of drug loading:* CIP-NE formulations were prepared using 0.1–0.3% *w*/*v* CIP loading. GS, PDI, ZP, and drug content of the CIP-NE formulations are shown in Table 4. CIP-O-NE-30 formulation showed GS, PDI, ZP, and drug content of 156.9 ± 1.2nm, 0.14 ± 0.01, −29.9 ± 0.6 mV, and 100.0 ± 5.0%, respectively, compared to 160.5 ± 1.9 nm, 0.23 ± 0.01, −24.5 ± 1.2 mV, and 102.1 ± 3.2%, respectively, for CIP-L-NE-30. CIP-O-NE formulations did not show a significant difference (*p* > 0.05) in GS, PDI, and ZP for different drug loads. Similarly, CIP-L-NE formulations also showed no significant difference in physical characteristics with different CIP drug loading. However, CIP-O-NE formulations showed a significantly lower globule size than CIP-L-NE formulations, which could be due to the branched triglyceride structure of LWL compared to a single-chain OA structure. In addition, CIP-O-NE formulation showed higher ZP than CIP-L-NE formulation, which could be due to free negative carboxyl groups of (OA in comparison to the esterified carboxyl group in LWL which consists of medium-chain triglycerides of caprylic (C_8_) and capric (C_10_) acids. This is consistent with an earlier published study [46]. Currently, commercial CIP ophthalmic solution available with 0.3% *w*/*v* drug loading. Therefore, CIP-O-NE-30 and CIP-L-NE-30 formulations were chosen as the optimized formulations, and they were further subjected to stability testing over three months (last time-point checked) at 4 °C, 25 °C, and 40 °C.

### 2.3. Stability Studies before Moist Heat Sterilization

**Figure 2:**
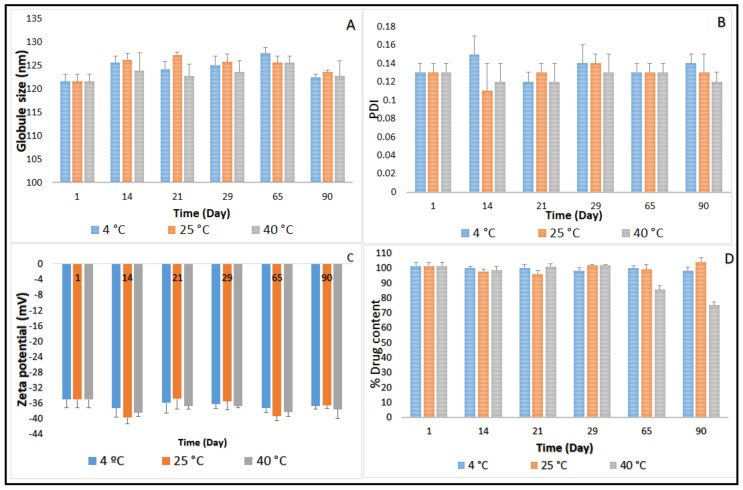
(**A**) Globule size, (**B**) polydispersity index, (**C**) zeta potential, and (**D**) ciprofloxacin content of CIP-O-NE-30 formulation over three-month storage at 4, 25 and 40 °C (mean ± SD, *n* = 3).

**Figure 3:**
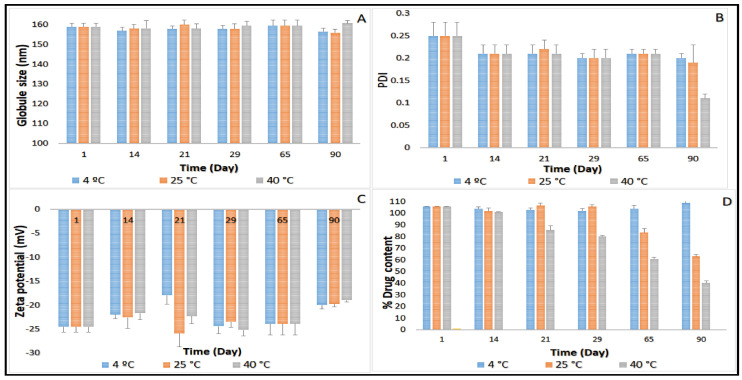
(**A**) Globule size, (**B**) polydispersity index, (**C**) zeta potential and (**D**) drug content of CIP-L-NE-30 formulation over three-month storage at 4, 25 and 40 °C (mean ± SD, *n* = 3).

Three-month stability data on GS, PDI, ZP, and drug content for CIP-O-NE-30 and CIP-L-NE-30 formulations stored at 4, 25 and 40 °C is presented in Figure 2 and Figure 3. GS of CIP-O-NE-30 formulation ranged from 121.6 to 127.2 nm, PDI ranged from 0.11 to 0.14, and ZP ranged from −34.9 to −39.7 mV at 25 °C. GS of CIP-L-NE-30 formulation ranged from 157.7 to 160.0 nm, PDI ranged from 0.20 to 0.25, and ZP ranged from −19.7 to −24.5 mV at 25 °C during the stability study. All NE formulations also showed narrow PDI, below 0.3. PDI of the lipid-based dispersions up to 0.5 was considered as homogeneous distribution [47]. Moreover, both NE formulations were stable for 90 days (last time-point tested) under the testing conditions regarding GS, PDI, and ZP, at 4, 25 and 40 °C. Brownian motion, induced by the small GS (20–200 nm) of NE formulations, provides stability against sedimentation or creaming, and keeps the diffusion rate higher than the sedimentation rate caused by the gravity force [48]. Therefore, both NE formulations did not show any phase separation, cracking, creaming, coalescence, or phase inversion for up to three months of storage (last time-point tested) at the three different storage conditions.

The main mechanism destabilizing NE is Ostwald ripening, which arises from emulsion polydispersity and the difference in solubility between small and large droplets [48]. Polymeric surfactants in O/W NE formulations, such as Tween^®^ 80 and Poloxamer 188, reduces the Ostwald ripening due to adsorption at the O/W interface, decreasing interfacial tension and increasing Gibbs dilatational elasticity [48,49]. It has been also reported that the addition of a dispersed phase insoluble surfactant stabilizes NE formulations against Ostwald ripening [48]. Moreover, the addition of a second surfactant with the same alkyl chain length and a higher degree of ethoxylation than the primary surfactant to an ethoxylated nonionic surfactant system led to a reduction in Ostwald ripening rate [48]. The observed physical stability for OA- and LWL-based NE formulations could be due to the absence of the Ostwald ripening behavior of NEs that could be attributed to small particle size, narrow PDI, and selected surfactant mixture composition.

Although there was no significant difference in drug content between the first and last time points during the stability study with the CIP-O-NE formulation at 4 and 25 °C, the drug content dropped from 101.5% to 85.4% after 65 days under the accelerated conditions (40 °C). The CIP-L-NE formulation also did not exhibit any significant difference in drug content between the first and last time points during the stability study at 4 °C; however, CIP content dropped from 105.6% to 83.4% after 65 days at room temperature and from 105.6% to 85.6% after 21 days at 40 °C. Future studies need to confirm the mechanism for the reduction in drug content at 40 °C. Based on the stability data, CIP-O-NE-30 formulation was selected for further evaluation with respect to moist heat sterilization, in vitro release, FTIR, and ex vivo transcorneal permeability.

### 2.4. Autoclavability of the Optimized NE Formulation

**Figure 4:**
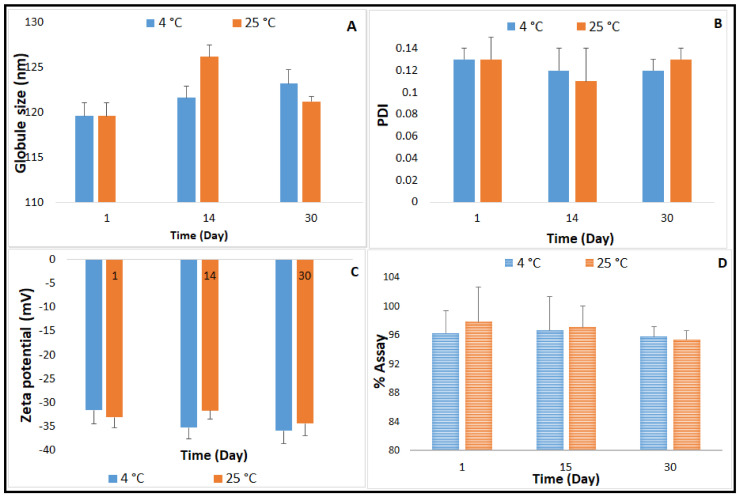
Effect of moist heat sterilization method on (**A**) globule size, (**B**) polydispersity index, (**C**) Zeta potential, and (**D**) drug content of CIP-O-NE-30 formulation over one-month storage at 4 and 25 °C (mean ± SD, *n* = 3).

NE formulations are good candidates for sterilization by filtration due to their small GS, which is less than the maximum nominal pore size of membrane filters used in this sterilization technique (0.22 or 220 nm). Filtration has been widely adopted as a sterilization method during the preparation of NE formulations specifically for thermolabile drugs; however, appropriate actions should be taken to avoid loss of solute by adsorption onto the filter, and to avoid the release of contaminants from the filter [50]. Furthermore, the aseptic filling of the sterilized solution is needed. Based on the evidence of autoclave process stability of CIP, reported in earlier studies [10], and the advantage as a terminal sterilization method in the final dosage form container, moist-heat sterilization was investigated for the optimized CIP-NE formulation [10]. CIP-O-NE-30 was selected for evaluating autoclave process-stability, based on the three-month stability study on the unsterilized formulations. Physicochemical characteristics post moist-heat sterilization is depicted in Figure 4. Following sterilization, CIP-O-NE-30 was able to preserve its characteristics. The autoclaved formulation remained stable at 4 and 25 °C; globule size (GS), polydispersity index (PDI), and zeta potential (ZP) did not show a significant change in comparison to pre-autoclaved formulation for one-month (last time-point tested). Sanela et al. reported that the physical stability parameters such as GS, PDI, and ZP of risperidone-containing NE formulations did not change after autoclaving compared to 0.22 µm-filtered NE formulations, and no significant change in these parameters was observed after one year of storage at 25 °C [51]. Arjun et al. also reported that there were no significant changes in GS, PDI, ZP, and drug content of the rebamipide containing NE formulation upon autoclaving [52]. After one-month storage, the autoclaved CIP-O-NE-30 formulation maintained the highly negative ZP values ranging from −31.6 to −35.9 mV and from −31.7 to −34.4 mV at 4 and 25 °C, respectively, reflecting adequately high negative surface charge for droplet–droplet repulsive interactions and, thus, improving NE stability.

### 2.5. FTIR Studies

**Figure 5:**
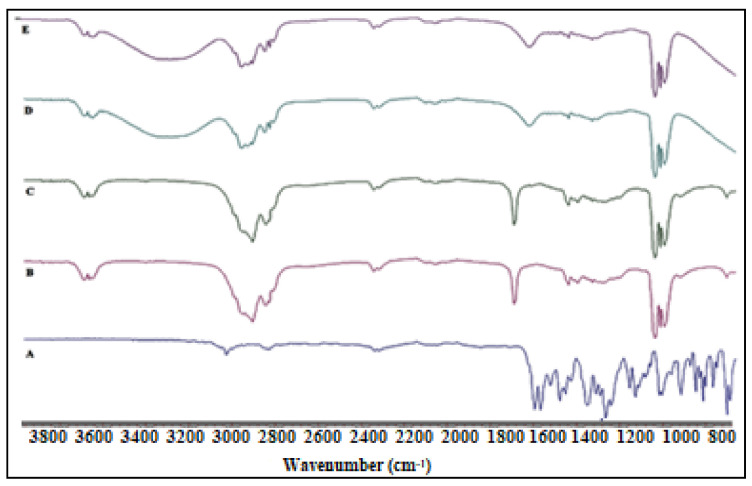
FTIR spectra of (**A**) ciprofloxacin, (**B**) oleic acid, (**C**) physical mixture (ciprofloxacin and oleic acid), (**D**) blank NE (CIP-O-NE-2), and (**E**) CIP-O-NE-30 formulation.

FTIR spectrum was collected for pure CIP, OA, physical mixture (CIP and OA), CIP-O-NE-2 (placebo NE), and CIP-O-NE-30 formulation (Figure 5) to investigate any incompatibility issues between the excipient and the drug. The FTIR spectrum of the pure OA shows a wide and intense band between 2880 cm^−1^ and 3006 cm^−1^ due to the O-H bond, which was centered at 2921 cm^−1^, and the C=O stretching band at 1708 cm^−1^ could be due to a dimeric OA. Furthermore, angular deformation outside the plane of the O-H bond gave a band at 1013 cm^−1^, which is characteristic of the dimeric OA [53]. CIP showed a carbonyl group stretching band at 1722 cm^−1^, a stretching vibration of the C-F bond at 1290 cm^−1^, and a C-H stretching vibration band of the phenyl ring at 3043 and 2918 cm^−1^ [54]. The absence of characteristic drug peaks in the physical mixture FTIR spectrum could be due to the high solubility of CIP in OA. Placebo and CIP containing NEs have a similar FTIR spectrum with a broad peak observed at 3200–3600 cm^−1^ which could be due to water O-H bond stretching which produces the continuous phase. The FTIR spectra did not show the characteristic CIP peaks in the blank and CIP-O-NE-30 formulations due to drug solubility within the oil globules; the observed peaks are from certain functional groups of the lipid excipients.

### 2.6. Transmission Electron Microscopy (TEM)

**Figure 6:**
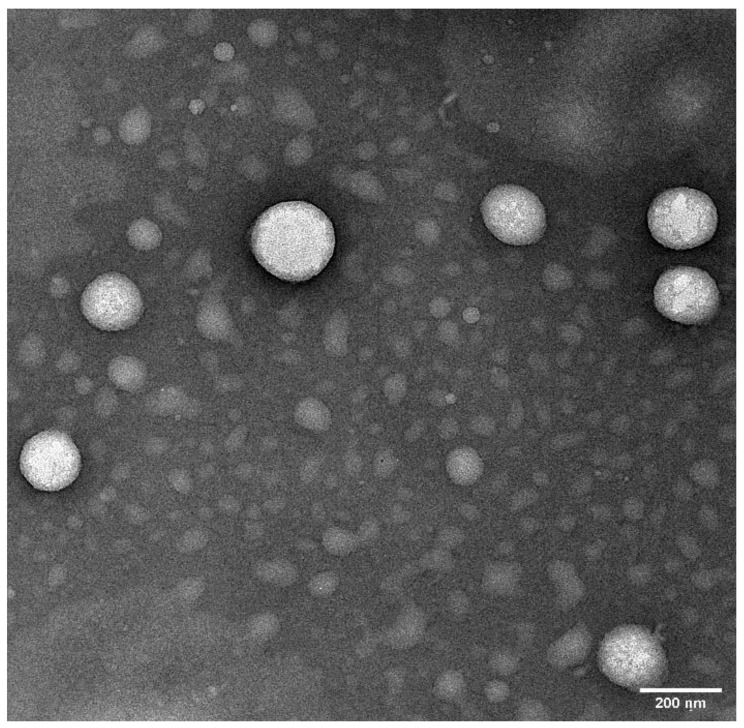
Transmission electron microscopic image of optimized ciprofloxacin nanoemulsion formulation. Image was taken at ×25 k magnification.

Surface morphology of CIP-O-NE-30 formulation was studied using TEM and the results are shown in Figure 6. The droplets were spherical in shape with size below 200 nm, correlating to the globule size measured by Zetasizer [55].

### 2.7. In Vitro Release Studies

**Figure 7:**
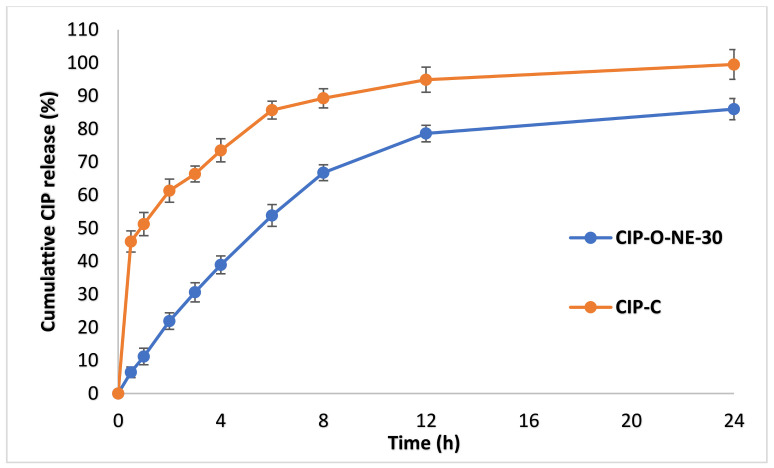
In vitro release of ciprofloxacin from CIP-O-NE-30 and commercial ciprofloxacin ophthalmic solution (CIP-C) formulation through Thermo Scientific™ Slide-A-Lyzer™ MINI Dialysis Device (10 K MWCO) (mean ± SD, *n* = 3).

The in vitro release of CIP from the CIP-C and optimized CIP-NE formulation is depicted in Figure 7. The overall cumulative percentage release was observed to be 99.5 ± 4.5% and 86.0 ± 3.2% from CIP-C and CIP-O-NE-30, respectively within the time course of the experiment (24 h). CIP-C solution released 80% of its CIP content during the first 5 h while CIP-NE formulation released this amount over 14 h.

The mathematical model that showed the highest R^2^ value was considered the best model to describe release kinetics. The highest value of the coefficient of determination (R^2^ = 0.9997) was observed for the Korsmeyer–Peppas model with a slope (n) value equal to 0.84, followed by the first-order (R^2^ = 0.9987), zero-order (R^2^ = 0.9929) and Higuchi’s (R^2^ = 0.9470) models for CIP-O-NE-30 formulation. The n-value indicated that drug release within the optimized NE formulation is diffusion controlled [56]. The sustained release behavior of NE formulation could be due to the release of hydrophobic drugs from O/W NE formulation involves many steps starting from partitioning (diffusion) of the drug from oil into surfactant and then into the aqueous phase [57].

### 2.8. Ex Vivo Transcorneal Permeation

**Figure 8:**
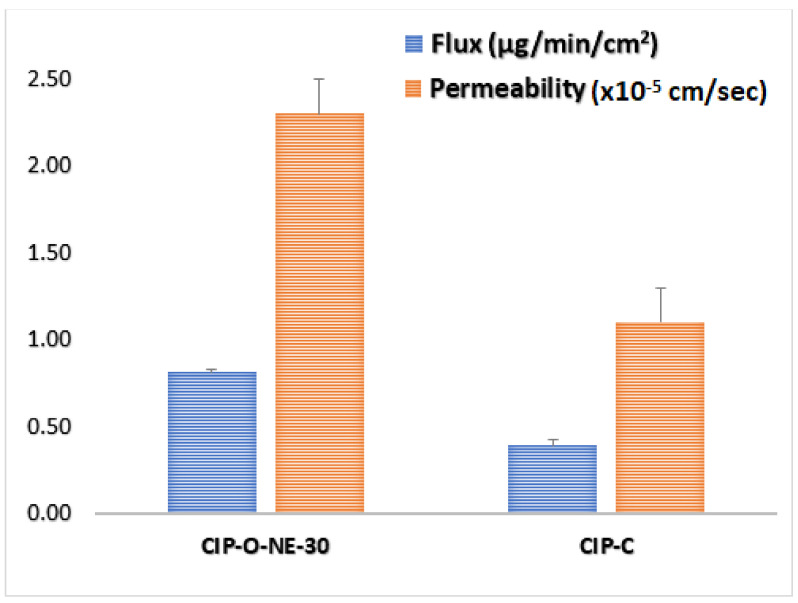
Transcorneal flux, and permeability of ciprofloxacin from CIP-O-NE-30 and ciprofloxacin ophthalmic solution (CIP-C) formulation through the isolated rabbit cornea (mean ± SD, *n* = 3)**.**

The rabbit cornea model is used for the evaluation of ocular drug delivery systems due to its close similarity to human cornea. Transcorneal flux and permeability of CIP from the formulations are shown in Table 5 and Figure 8. The transcorneal flux of CIP from CIP-O-NE-30 and CIP-C formulation was 0.81 ± 0.02 and 0.39 ± 0.03 μg/min/cm^2^, respectively. Similarly, the transcorneal permeability coefficient of CIP from CIP-O-NE-30 and CIP-C formulation was found to be 2.3 ± 0.05 × 10^−5^ and 1.1 ± 0.09 × 10^−5^ cm/s respectively.

The reasons for enhanced corneal permeation from optimized CIP-NE formulation could be due to the following reasons: NE formulations are capable of enhancing drug permeation through the cornea due to the presence of surfactants and cosurfactants which increase the membrane permeability, thereby increasing drug uptake [32]. In addition, the nano-size range (100–200 nm) of the NE formulations can also be internalized by receptor-mediated endocytosis uptake mechanisms through the corneal cells [10,11]. Additionally, small globules have a superior chance to cover a large surface area, allowing enhanced penetration of drugs [33]. Moreover, OA has been reported to have potential clinical benefits by improving ophthalmic drug delivery of both lipophilic and hydrophilic compounds [58]. There are no clear proposed mechanisms for the penetration-enhancing effect of OA, but OA may produce ultrastructure changes in corneal epithelium, creating pathways for drugs by disrupting the highly ordered lipid bilayer, leading to the fluidization of cell membrane lipids.

The corneal epithelium and endothelium layers limit water transfer to the corneal stroma, and these two layers are responsible for the corneal hydration level [59]. The normal hydration level of the cornea is from 76% to 80%. However, epithelial or endothelial layer damage allows water to enter the stroma leading to corneal edema. Corneal hydration studies have been considered as a good indicator for corneal damage, and increasing hydration level to 83% or more indicates corneal damage [59]. Recent studies reported that physiological porcine and human corneal hydration was maintained following 24 h immersion in 3.25% and 4.25% *w*/*v* Poloxamer 188 [60]. In the current study, the concentration of Poloxamer 188 (0.2% *w*/*v*) used for the preparation of the NE formulations was much lower. OA concentrations of 0.02–0.1% have also been reported to be safe based on corneal hydration studies after ocular application of OA containing NEs. Higher OA concentrations (3% *w*/*v*) have also been reported to be safe on ocular instillation [10]. The concentration of OA (5% *w*/*v*) used in this study, however, is higher than that in the earlier studies and thus additional investigations with respect to corneal hydration, in vivo ocular tolerance, and histology studies will be required to assess corneal tissue integrity after topical instillation of the optimized NE formulation. The highest concentration of Tween^®^ 80 according to the Food and Drug Administration (FDA) inactive ingredient database for approved ophthalmic NE drug products is 4% *w*/*w* which is two-times the concentration that was used in the preparation of the optimized CIP-NE formulations [61].

A review of the literature indicates that the highest transcorneal permeability of CIP was achieved by loading CIP into a liposomal formulation which increased the ocular bioavailability of CIP by almost 4-fold compared with commercial CIP eye drops [35]. Sai et al. also reported that the transcorneal flux of CIP from their optimized Pegylated nanostructured lipid carrier’s formulation was almost 3-folds higher than commercial eye drop [10]. Moreover, Youssef et al. reported that the transcorneal flux and permeability increased by 4 and 3.5-fold from CIP-containing nanostructured lipid carrier’s formulation, respectively, when compared to the control solution [11]. In this study, the NE formulation exhibited a 2.1-fold enhancement in corneal flux and permeability. The NE formulation presents several advantages including patient comfort, cost, and ease of manufacturability, compared to the other formulations [62,63].

## 3. Materials and Methods

### 3.1. Materials

CIP was purchased from Sigma Aldrich (St. Louis, MO, USA). Labrafac^®^ Lipophile WL 1349 (LWL) was a generous gift sample from Gattefossé (Paramus, NJ, USA). Oleic acid (OA), Tween^®^ 80, Poloxamer 188, and 0.5 mL cup-like design Thermo Scientific™ Slide-A-Lyzer™ MINI Dialysis Device (10 K molecular weight cutoff) were obtained from Fischer Scientific (Hampton, NH, USA). Other chemicals and glassware required for the project such as high performance liquid chromatography (HPLC) grade solvents, scintillation vials, centrifuge tubes, HPLC vials were acquired from Fischer Scientific (Hampton, NH, USA) and Gattefossé (Paramus, NJ, USA). Whole eyes of male albino New Zealand rabbits were purchased from Pel-Freez Biologicals (Rogers, AR).

### 3.2. Methods

#### 3.2.1. Screening of Oils

The solubility of CIP in various oils was determined by adding 10 mg of drug in 100 mg of the oils (olive oil, castor oil, soybean oil, sesame oil, OA, Miglyol^®^ 829, Maisine^®^ CC, Captex^®^ 355 EP, LWL, and Capryol 90^TM^) in 3-mL-capacity glass vials, and then mixing using a vortex mixer. The mixture was then heated at 80 ± 2 °C, under continuous magnetic stirring at 2000 rpm, for 5 min, following which the CIP-oil mixtures were cooled. The mixtures were then visually examined for CIP precipitation and oils that did not show any precipitation were selected.

#### 3.2.2. Quantification of CIP by HPLC

Quantitative determination of CIP was performed based on an earlier published HPLC method after some modification and subsequent validation [64]. The HPLC system consisted of a Waters 717 plus auto-sampler coupled with a Waters 2487 Dual λ Absorbance UV detector, a Waters 600 controller pump, and an Agilent 3395 Integrator. Chromatographic separation was achieved on a reverse-phase C_18_ column, Phenomenex Luna^®^ (5 μ, 250 × 4.6 mm) using a mobile phase consisting of acetonitrile and 0.025 M phosphate buffer pH 2.4 (30:70 % *v*/*v*) at a flow rate of 1 mL/min with UV detection λ at 290 nm and absorbance units full scale (AUFS) 1.000. The analysis was performed at 25 °C, the injection volume was 20 μL, and the pH was adjusted by the addition of sodium hydroxide solution. The method was found to be linear over the concentration range of 1–30 μg/mL. The modified method was found to be precise and accurate with a limit of detection (LOD) and limit of quantitation (LOQ) of 0.4 and 1.2 µg/mL, respectively.

#### 3.2.3. Preparation of CIP-NE Formulations

Oil-in-water (O/W) type NE formulations were prepared by hot homogenization followed by ultra-probe sonication [10]. The aqueous phase was prepared by adding surfactants (Tween^®^ 80 and Poloxamer 188) and glycerin (2.25% *w*/*v*) to double distilled Milli-Q water and heated to 80 ± 2 °C. The aqueous phase was then added to the oil phase, maintained at the same temperature, under continuous magnetic stirring to form a primary emulsion (2000 rpm, 5 min). The dispersed phase consisted of CIP dissolved in OA or LWL. The primary emulsion was further emulsified at 11,000 rpm for 5 min using a T25 digital Ultra-Turrax (IKA, Germany) at 65 ± 2 °C, to form a hot emulsion. The hot emulsion was then subjected to probe sonication at 40% amplitude with 3 mm stepped microtip at 500 watts power supply and 115 volts for 10 min with a 10-sec pulse ON and 10-sec pulse OFF using Sonics Vibra Cell Sonicator (Newtown, CT, USA) to form NE.

#### 3.2.4. Control Formulation (CIP-C)

Ciprofloxacin hydrochloride ophthalmic solution, 0.3% as a base (Alcon Laboratories, Fort Worth, TX, USA; Lot # 295240F).

#### 3.2.5. Characterization of CIP-NE

##### Measurement of Globule Size (GS), Polydispersity Index (PDI), and Zeta Potential (ZP)

GS, PDI, and ZP of the prepared CIP-NE formulations were determined by photon correlation spectroscopy using a Zetasizer Nano ZS Zen3600 (Malvern Instruments, MA, USA) at 25 °C in disposable, folded, clear capillary cells. GS and PDI measurements were attained using a helium-neon laser, and the data were analyzed based on the volume distribution. The samples were diluted 100 times with filtered bi-distilled water and evaluated for GS, PDI, and ZP in triplicate at 25 °C [65].

##### Drug Content

For drug content analysis, 10 μL formulation and 990 μL solvent mixture (0.1N HCl and 200-proof alcohol (1:1)) were mixed and centrifuged using AccuSpin 17R centrifuge (Fisher Scientific, Waltham, MA, USA) for 20 min at 13,000 rpm. The supernatant was collected and drug content in the supernatant was analyzed using HPLC following a 2-fold dilution with the mobile phase.

##### Physicochemical Stability

Stability study for test formulations was initiated under refrigerated (4 ± 2 °C), room temperature (25 ± 2 °C), and accelerated (40 ± 2 °C) storage conditions. The formulation was evaluated for physical instability issues—phase separation, cracking, creaming, coalescence, or phase inversion, change in GS, PDI, ZP—and change in drug content at predetermined time intervals for up to three months.

##### Terminal Moist Heat Sterilization and Stability Assessment of CIP-NE Formulation

Test formulation was prepared and added to appropriately labeled glass vials, affixed with sterilization indicator tapes, and subjected to moist heat sterilization (121 °C for 15 min under 15 psi) (Tuttauer, Heidolph, Germany). The sterilization cycle was validated through the change in the color of the indicator tapes fixed on the glass vials. Following autoclaving, formulations were stored at 4 ± 2 and 25 ± 2 °C for one month (last-time point checked) and evaluated in the same way as the pre-autoclaved samples.

#### 3.2.6. Fourier Transform Infrared Spectroscopy (FTIR)

The interaction between the drug and other formulation excipients was evaluated using FTIR spectroscopy. The bench was equipped with an ATR (Pike Technologies MIRacle ATR, Madison, WI) that was fitted with a single bounce diamond-coated ZnSe internal reflection element. The scanning range was 800–4000 cm^−1^.

#### 3.2.7. Surface Morphology—Transmission Electron Microscopy (TEM)

Surface morphology of the optimized CIP-NE formulation was observed through TEM [55,66]. The sample was analyzed through negative staining protocol. The grid is placed over a 20 µL drop of the sample solution for 1 min. The excess sample is drawn-off the grid with the help of a filter paper. The grid is washed briefly by dipping it in distilled water and removing the excess water from grid by using filter paper. The grid is stained immediately using UranyLess for 1 min and then allowed to dry for a few minutes, followed by imaging using JEOL 1400-Flash TEM. All the images are taken at 25K times magnification.

#### 3.2.8. In Vitro Release Studies

Isotonic phosphate-buffered saline (IPBS; pH 7.4) containing 2.5% *w*/*v* randomly methylated beta-cyclodextrin (RMβCD) was used as the receiver medium for in vitro release and ex vivo transcorneal permeation studies based on earlier reported CIP solubility studies in different dissolution media [10,11]. A 0.5 mL cup-like design Thermo Scientific™ Slide-A-Lyzer™ MINI dialysis Device (10 k molecular weight cutoff) was used to study the drug release from the test and commercial formulations. Briefly, 200 µL of the formulations were added to the dialysis device, which was considered as the donor compartment, mounted on the top of 20 mL scintillation glass vials (receiver compartment), placed on a multi-stationed magnetic stirrer (IKA, USA) at 500 rpm, programmed at static conditions. The receiver compartment, containing 20 mL receiver medium was maintained under continuous magnetic stirring at 34 °C. Samples (1 mL) were collected at pre-determined time intervals over 24 h, and an equivalent volume of freshly prepared receiver medium was replenished. Donor CIP concentration was maintained at 0.3% *w*/*v* for all test and control formulations. Samples were analyzed using HPLC.

CIP release data were kinetically analyzed to study the possible mechanisms of drug release using Kinet DS3 software (Department of Pharmaceutical Technology and Biopharmaceutics, Faculty of Pharmacy, Jagiellonian University). Linear regression equations were used, and the coefficient of determination (R^2^) was calculated. Data for regression analysis were processed using the statistical function of Microsoft^®^ (MS) office excel (Office365, 2019, USA). To study the mechanism of drug release from NE, Zero-order, First-order, Higuchi, and Korsmeyer–Peppas equations [67,68] were chosen as mathematical models to characterize the in vitro release profile. These models are commonly used to describe the drug release pattern from drug delivery systems when the drug release mechanism is not clear or when there is more than one type of release phenomenon involved. The model that gave the highest coefficient of determination (R^2^) was deemed to be the most appropriate kinetic model to describe CIP release.

#### 3.2.9. Ex Vivo Transcorneal Permeation Studies

Ex vivo transcorneal permeation studies for test and control formulations were performed on corneas isolated from rabbit whole eyes, shipped from Pel-Freez Biologicals (Rogers, AR, USA). The eyes were preserved in Hanks’ balanced salt solution in ice and shipped overnight. The corneas were carefully excised and used for the permeation studies immediately upon their arrival. The isolated corneas were washed in IPBS solution, pH 7.4. The cornea was clamped in between the two halves of the vertical diffusion cells (PermeGear^®^, Inc., Hellertown, PA, USA) with the epithelial surface facing the donor half-cell containing 200 µL of the test or control formulation. Donor CIP concentration was maintained at 0.3% *w*/*v* for all test and control formulations. The content of the receiver compartment (five milliliters of 2.5% solution of RMβCD in IPBS; pH 7.4) was maintained under continuous magnetic stirring at 34 °C with the help of a circulating water bath. Samples (600 µL) were collected from the receiver chamber at pre-determined time points and replaced with an equal volume of the receiver medium. The samples were quantified using HPLC. The cumulative amount of CIP permeated (*Q_n_*), steady-state flux (*J_ss_*), and transcorneal permeability (*P_eff_*), across the isolated rabbit cornea, were calculated to study CIP transcorneal permeation. All the studies were conducted in triplicate. *Q_n_* was calculated using the following equation:

(1) $$Q_{n}=V_{r}C_{r\left(n\right)}+\overset{x=n}{\underset{x=1}{\sum }}V_{s\left(x-1\right)}C_{r\left(x-1\right)}$$

where, *n* is sampling time point; *V_r_* is the volume in the receptor chamber (mL), *V_s_* is the volume of the aliquot withdrawn at the nth time point (mL) and *C_r_*_(*n*)_ is CIP concentration in the receptor chamber medium at the nth time point (µg/mL).

The rate of transcorneal permeation (*dQ*/*dt*) was calculated using the slope of the (*Q_n_*) of CIP transported versus time plot. *J_ss_* of CIP was determined using the following equation:

*J_ss_* = (*dQ*/*dt*)/*A* (2)

where, *Q* is the cumulative amount of CIP transported and *A* is the corneal permeation surface area (0.636 cm^2^).

The permeability of CIP was calculated by the following equation:

*P_eff_* = *J_ss_*/*C*_0_ (3)

where, *C*_0_ is the initial donor concentration of CIP.

#### 3.2.10. Statistical Analysis

The results are presented as mean ± standard deviation. GS, PDI, ZP, drug content, transcorneal flux, and permeability were treated statistically using a one-way analysis of variance (ANOVA) with SPSS (IBM SPSS Statistics software, SPSS 26, Armonk, NY, USA). When there was a statistically significant difference, a post-hoc Tukey-HSD (Honestly Significant Difference) test was performed. A statistically significant difference was observed at a *p*-value less than 0.05 (*p* < 0.05).

## 4. Conclusions

CIP loaded NE formulations were successfully optimized, autoclaved, and evaluated for stability over three months at three different storage conditions. Optimized CIP-NE (CIP-O-NE-30) formulation was stable at 4 and 25 °C for at least one-month post moist heat sterilization. The in vitro release experiments revealed a sustained release profile for CIP from the CIP-NE formulation. Moreover, the ex vivo studies showed a 2.1-fold improvement in transcorneal permeability and flux when compared to the control solution. In vivo ocular biodistribution and efficacy evaluation in a rabbit BK model are additional studies that are needed for the formulation to be developed into an ocular dosage form for the treatment of BK. In summary, the optimized CIP-NE formulation could be a promising drug delivery platform for the effective delivery of CIP in the treatment of BK and several other ocular bacterial infections.

## Figures and Tables

**Table:** 

Formulation	Tween^®^ 80 (% *w*/*v*)	Formulation	Tween^®^ 80 (% *w*/*v*)
O-NE-0.75	0.75	L-NE-0.75	0.75
O-NE-1	1	L-NE-1	1
O-NE-1.5	1.5	L-NE-1.5	1.5
O-NE-2	2	L-NE-2	2

O-NE and L-NE placebo formulations were prepared with oleic acid (5% *w*/*v*) and Labrafac^®^ Lipophile WL 1349 (5% *w*/*v*), respectively. All formulations contain 0.2% *w*/*v* of poloxamer 188 and 2.25% *w*/*v* glycerin.

**Table 1:** Oil screening study for ciprofloxacin.

Oil	Solubility	Oil	Solubility
Soybean oil	(−)	Miglyol^®^ 829	(−)
Captex^®^ 355 EP	(−)	Castor oil	(−)
Oleic acid	(+)	Labrafac^®^ Lipophile WL 1349	(+)
Sesame oil	(−)	Capryol 90^TM^	(−)
Maisine^®^ CC	(−)	Olive oil	(−)

(+): CIP is soluble in the oil and does not precipitate on cooling; (−): CIP is either soluble in the oil, but precipitates on cooling or is insoluble in the oil.

**Table 2:** Composition of ciprofloxacin nanoemulsion formulations.

Formulation Composition (% *w*/*v*)	CIP-O-NE-10	CIP-O-NE-20	CIP-O-NE-30	CIP-L-NE-10	CIP-L-NE-20	CIP-L-NE-30
Ciprofloxacin	0.1	0.2	0.3	0.1	0.2	0.3
Oleic acid	5	5	5	-	-	-
Labrafac^®^ Lipophile WL 1349	-	-	-	5	5	5
Tween^®^ 80	2	2	2	2	2	2
Poloxamer 188	0.2	0.2	0.2	0.2	0.2	0.2
Glycerin	2.25	2.25	2.25	2.25	2.25	2.25
Water up to	10 mL	10 mL	10 mL	10 mL	10 mL	10 mL

**Table 3:** Effect of homogenization speed (5 min) and probe sonication (10 min) on globule size and polydispersity index of two different placebo nanoemulsion (NE) formulations (mean ± SD, *n* = 3).

Formulation	Homogenization Speed (rpm)	With 10 min Sonication		Without Sonication	
		Globule Size (nm)	PDI	Globule Size (nm)	PDI
**O-NE-2**	9000	225.5 ± 5.1	0.15 ± 0.04	741.3 ± 60.5	0.61 ± 0.09
	10,000	187.8 ± 3.7	0.11 ± 0.04	551.3 ± 75.2	0.53 ± 0.04
	11,000	96.3 ± 2.5	0.13 ± 0.02	395.2 ± 32.7	0.43 ± 0.05
**L-NE-2**	9000	283.5 ± 3.6	0.22 ± 0.02	1098.6 ± 93.3	0.92 ± 0.05
	10,000	234.7 ± 4.2	0.22 ± 0.02	760.1 ± 74.5	0.71 ± 0.06
	11,000	132.2 ± 1.9	0.23 ± 0.03	488.2 ± 70.4	0.59 ± 0.02

O-NE-2 and L-NE-2 prepared as per the composition of CIP-O-NE-20 and CIP-L-NE-20 excluding the CIP.

**Table 4:** Globule size, polydispersity index, zeta potential, and drug content of different ciprofloxacin-containing NE formulations (mean ± SD, *n* = 3).

Formulation	CIP-O-NE-10	CIP-O-NE-20	CIP-O-NE-30	CIP-L-NE-10	CIP-L-NE-20	CIP-L-NE-30
PS (nm)	134.9 ± 2.9	140.5 ± 1.3	156.9 ± 1.2	156.6 ± 2.3	158.9 ± 1.6	160.5 ± 1.9
PDI	0.12 ± 0.07	0.15 ± 0.03	0.13 ± 0.01	0.20 ± 0.02	0.23 ± 0.01	0.23 ± 0.01
ZP (mV)	−25.13 ± 0.9	−27.06 ± 0.4	−29.9 ± 0.55	−21.9 ± 2.1	−23.2 ± 1.5	−24.5 ± 1.2
Drug content (%)	95.8 ± 1.1	97.7 ± 2.6	100.0 ± 5.1	94.9 ± 2.2	95.8 ± 2.1	102.1 ± 3.2

**Table 5:** Flux and permeability of ciprofloxacin from CIP-O-NE-30 and commercial ciprofloxacin ophthalmic solution (CIP-C) formulation across isolated rabbit cornea (mean ± SD, *n* = 3).

Formulation	Flux (µg/min/cm^2^)	Permeability (×10^−5^ cm/s)	Folds Enhancement with CIP-C	
			Flux	P
**CIP-C**	0.39 ± 0.01	1.1 ± 0.09	-	-
**CIP-O-NE-30**	0.81 ± 0.03 ^#^	2.3 ± 0.05 ^#^	2.1	2.1

^#^ indicates statistically significant at *p* < 0.05 compared with CIP-C formulation.

## Data Availability

The data presented in this study are available on request from the corresponding author.
